# The value of performance-based tests in the assessment of patients with fibromyalgia: a construct validity and comprehensiveness analysis of a standardized test battery

**DOI:** 10.3389/fresc.2026.1947752

**Published:** 2026-09-11

**Authors:** José Luis Socorro-Cumplido, Joaquim Chaler, Miriam Almirall, Judith Sánchez-Raya, Josep Blanch-Rubió, Maria José Castro, Maria Giné-Garriga, Patricia Launois, Tamara Rodríguez-Araya, Anna Arias Gassol, Geert Crombez, Raimon Milà Villarroel, Blanca Roman-Viñas

**Affiliations:** 1 Blanquerna School of Psychology, Education and Sports Sciences, Universitat Ramon Llull, Barcelona, Spain; 2 Physical Medicine and Rehabilitation Department, Hospital Egarsat, Barcelona, Spain; 3 EUSES University School of Health and Sports, University of Girona–University of Barcelona, L’Hospitalet de Llobregat, Spain; 4 Rheumatology Department, University Hospital Vall D’Hebron, Barcelona, Spain; 5 Physical Medicine and Rehabilitation, Hospital Vall D’Hebron, Barcelona, Spain; 6 Rheumatology Department, Hospital del Mar, Barcelona, Spain; 7 Clinical Expertise Unit for Central Sensitization Syndromes, Hospital del Mar, Barcelona, Spain; 8 Blanquerna School of Health Sciences, Universitat Ramon Llull, Barcelona, Spain; 9 Rheumatology Department, Hospital Clínic, Barcelona, Spain; 10 Department of Experimental-Clinical and Health Psychology, Ghent University, Ghent, Belgium; 11 EUSES University School of Health and Sports, University of Girona, Salt, Spain

**Keywords:** fibromyalgia, functional assessment, international classification of functioning disability and health (IFC), objective physical performance, patient-reported outcome measures (PROMs)

## Abstract

**Background:**

Comprehensive assessment of functioning in fibromyalgia (FM) requires the integration of objective and subjective outcome measures. Although performance-based tests (PBTs) are increasingly recommended for evaluating physical function, evidence supporting their construct validity within a standardized multidimensional assessment framework remains limited. Furthermore, the complementary contribution of accelerometer-derived physical activity measures has not been fully established.

**Objective:**

To evaluate the construct validity and comprehensiveness of a standardized assessment battery integrating PBTs, patient-reported outcome measures (PROMs), and accelerometer-derived physical activity measures in women with FM within an International Classification of Functioning, Disability and Health (ICF)-based framework.

**Methods:**

A cross-sectional study included 87 women with FM recruited from four tertiary hospitals. Participants completed four PBTs (6-minute walk test, handgrip strength test, 8 foot up and go test, and Berg Balance Scale), accelerometer-based physical activity assessment, and validated Spanish PROMs assessing physical functioning, emotional distress, and pain impact and symptom burden. Construct validity was evaluated by testing predefined hypotheses for convergent and discriminant validity using Spearman correlations. Exploratory factor analysis (EFA) was conducted to examine the latent multidimensional structure of the proposed assessment framework.

**Results:**

The EFA identified four latent constructs corresponding to physical functioning, activity behavior, emotional distress, and pain impact and symptom burden, explaining 67.1% of the total variance. The physical functioning construct accounted for the largest proportion of explained variance (22.6%), followed by activity behavior (18.6%), emotional distress (13.3%), and pain impact and symptom burden (12.7%). Correlation analyses supported the predefined hypotheses, demonstrating moderate to strong within domain associations and generally weak to moderate between domain correlations, confirming both convergent and discriminant validity. ICF mapping showed that the proposed battery provided broad coverage of key body functions and activity domains, while accelerometer-derived variables emerged as an independent construct, supporting their complementary value beyond PBTs and PROMs.

**Conclusion:**

The proposed standardized assessment battery demonstrates strong construct validity and provides a comprehensive ICF-based evaluation of functioning in women with FM. The combination of PBTs, accelerometry, and PROMs offers a multidimensional characterization of functioning that may improve functional assessment, individualized rehabilitation planning, and treatment monitoring.

**Clinical Trial Registration:**

https://clinicaltrials.gov/study/NCT06819930, identifier NCT06819930

## 1 Introduction

Fibromyalgia (FM) is a chronic disease characterized by widespread musculoskeletal pain accompanied by non-restorative sleep, fatigue, impaired cognitive function and often psychological symptoms, leading to a substantial impairment in functioning and health-related quality of life (HRQoL) (1, 2). Prevalence estimates vary across diagnostic definitions and settings, ranging from 0.2% to 6.6%; nevertheless, contemporary syntheses consistently depict FM as a common disease worldwide, with much higher rates in women (3, 4). Beyond symptoms, FM carries significant major societal consequences, including reduced quality of life, work disability, increased healthcare utilization, and substantial direct and indirect costs (5, 6). FM is currently diagnosed according to the American College of Rheumatology (ACR) criteria, which are based on self-reported pain distribution and symptom severity. Although these criteria facilitate clinical diagnosis, they do not include objective indicators of physical function or functional impairment, underscoring the need for reliable biomarkers and objective assessment tools (7). Additionally, clinical characterization and monitoring of FM rely heavily on patient-reported outcome measures (PROMs). While these instruments are essential for capturing the patient's subjective experience and disease impact, their interpretation is influenced by the complex biopsychosocial nature of the disease. Perceived functioning may be affected by symptom appraisal, emotional distress, coping strategies, and contextual factors, which do not necessarily correspond directly to objectively measurable functional performance. Consequently, PROMs may not fully reflect actual functioning outcomes and should ideally be complemented by objective assessments of physical capacity and performance (8).

A comprehensive multidimensional assessment is mandatory in FM including several functioning domains. Within the International Classification of Functioning, Disability and Health (ICF), “functioning” is conceptualized as a dynamic interaction between health condition, personal factors, and environmental context (9). This framework implies that a rigorous evaluation of functioning should triangulate across complementary domains: (i) capacity (what a person can do under standardized conditions), (ii) performance (what a person does in daily life), and (iii) perceived impact (how limitations are experienced and reported). Performance-based tests (PBTs) quantify capacity under controlled conditions and are therefore conceptually aligned with ICF activities and body functions domains (10, 11). The most common PBTs used to evaluate FM patients such as the 6-minute walk test (6MWT), handgrip strength test (HST), and the 8 foot up and go test (8FUGT) capture partially distinct body functions and activities such as exercise tolerance (b455), muscle power (b730), walking capacity (d450), moving around (d455) and changing basic body position (d410) (12–14); all of them are categories gathered within the ICF-based “core set” for Chronic Widespread Pain (CWP) (15).

While PBTs assess physical capacity under standardized conditions, accelerometry objectively quantifies habitual physical activity (PA) and sedentary behavior in free-living environments, thereby providing complementary information on patients real-world functioning (16). Even though PBTs and actigraphy/accelerometry are highly recommended in chronic pain patients both for research and clinical purposes (11), the standardization of patients with FM assessment is far from being implemented. It is noteworthy that a recent article on multidimensional assessment in such patients just included one PBT (6MWT) in the set of measurements (17). Additionally, exercise and meditative movement therapies demonstrate a high effectiveness in FM patients with a high level of evidence (18). PBTs and accelerometry directly measure the results of such approaches (e.g., exercise tolerance, strength or balance improvements) and might be helpful in tailoring a specific training program for the patients targeting specific needs, and capturing the effectiveness of any therapy regime.

Thus, it may be stated that the need to include valid PBTs in the context of comprehensive functioning assessment of persons with FM is strongly advocated (11). The Consensus-based Standards for the selection of health Measurement Instruments (COSMIN) defines construct validity as the degree to which scores behave consistently with *a priori* hypotheses regarding relationships with other measures and differences between relevant groups (19, 20). In FM, while reliability and feasibility of PBTs (including 6MWT, HST and 8FUGT) has been reported (11, 18–20), comprehensive construct validity investigations remain comparatively scarce and heterogeneous, limiting interpretability and cross-study comparability (21).

Therefore, the main objective of this study was to evaluate the construct validity and comprehensiveness of a standardized test battery including PBTs in women with FM. Specifically, we aimed to (1) examine the convergent validity by assessing the associations between PBTs based outcomes and PROM-derived indicators of perceived physical functioning and disease impact, as well as accelerometer-derived measures of habitual physical activity; to (2) assess the discriminant validity by analyzing the relationships between PBTs outcomes and conceptually-derived distinct domains, including emotional distress, symptom burden, and activity behavior; and to (3) examine the comprehensiveness of the proposed battery by analyzing the factor loads of the domains depicted by the assessment tools (physical functioning, activity behavior, emotional distress, pain impact and symptom burden). We hypothesized that both PBTs and accelerometry would demonstrate an adequate construct validity and help capture the substantial variability observed in multidimensional FM assessment. Through this multilevel approach, we sought to establish the theoretical coherence and clinical usefulness of objective capacity, PBTs based measures within an ICF-based, biopsychosocial model of functioning assessment in patients with FM.

## 2 Materials and methods

### 2.1 Study design

An observational cross-sectional study was conducted to evaluate the construct validity of a standardized battery of PBTs in women with FM. The study was designed in accordance with the COSMIN guidelines.

The selected PBTs (6MWT, HST, and the 8FUGT) assess key domains of physical functioning commonly impaired in FM (exercise tolerance, muscle power, and changing and maintaining basic body position) all included within the ICF framework (22). In addition, these tests have demonstrated feasibility, safety, and appropriate psychometric properties in patients with chronic pain, including individuals with FM, and they are widely used in both clinical and research settings due to their clinical interpretability and minimal equipment requirements (23). The Berg Balance Scale (BBS) was added to reinforce balance assessment and due to its good reliability in patients with FM (24–26).

To enable a comprehensive evaluation of construct validity, objective physical capacity measures (PBTs) were collected alongside PROMs and accelerometer-derived indicators of habitual PA. All variables were obtained within a single assessment period using standardized protocols. PBTs were administered under controlled conditions, while PROMs and PA measures were collected using validated instruments and procedures.

Overall, this study design allowed for a multilevel assessment of functioning which was aligned with the proposal of a comprehensive, feasible set of measurements in FM, integrating objective capacity, perceived impact, and real-world activity behavior, in line with a biopsychosocial and ICF-based conceptualization of the condition through previously proposed core sets.

### 2.2 Participants

Patients with FM were recruited from 4 hospitals in Barcelona (Hospital Clínic, Hospital Egarsat, Hospital del Mar, and Hospital de la Vall d'Hebron) between February 2024 and April 2025 by e-mail or telephone. Those who agreed to participate contacted the research team via email and were screened for eligibility before inclusion. The participants fulfilled the following inclusion criteria: (a) female between 30 and 65 years old, (b) diagnosed with FM by a rheumatologist according to the ACR criteria (7), (c) understand the PBT protocols, and (d) be able to communicate effectively with the study researchers. Exclusion criteria were: (a) to show illiteracy and/or lack of understanding of Spanish, (b) to have severe psychiatric and/or psychological disorders, (c) to have other rheumatological, autoimmune, neurological, or musculoskeletal conditions that could affect physical performance (e.g., inflammatory arthritis, systemic autoimmune diseases, neurological disorders affecting mobility), and (d) patients who refused to participate in the study.

### 2.3 Ethical approval

This study was conducted in accordance with the ethical principles of the Declaration of Helsinki and in compliance with applicable national regulations governing biomedical research. Written informed consent was obtained from all participants prior to their enrollment. Ethical approval was obtained from all participating institutions. The protocol was reviewed and approved by the Clinical Research Ethics Committee of Parc de Salut Mar (CEIm-PSMAR; reference 2024/11477/I), the Clinical Research Ethics Committee of Hospital Clínic de Barcelona (Reg. HCB/2024/1032), the Ethics Committee of the Fundació Assistencial Mútua Terrassa (reference P/21-082), and the Ethics and Research Committee of Universitat Ramon Llull (reference 2223019D). Each committee evaluated and approved the protocol, the patient information sheet, and the informed consent form. Written informed consent was obtained from all participants prior to inclusion. The study was registered in ClinicalTrials.gov under the identifier NCT06819930.

### 2.4 Assessment procedures

#### 2.4.1 Anthropometric and sociodemographic data

Height (m) was measured using a stadiometer (Seca® 22, Hamburg, Germany) and weight (kg) with a scale (InBody ® 720, Biospace, Seoul, Korea). Body mass index (BMI) was calculated as weight (kg) divided by height squared (m2). Participants were classified according to the World Health Organization (WHO) BMI categories as underweight (< 18.5 kg/m^2^), normal weight (18.5–24.9 kg/m^2^), overweight (≥ 25.0 kg/m^2^), and obesity (≥ 30.0 kg/m^2^) (27). Sociodemographic data were collected including age, ethnicity, marital status, educational level, and work status (actively employed, paid sick leave and or claiming disability, recognized disability).

#### 2.4.2 Questionnaires

The Spanish versions of the Widespread Pain Index (WPI) and Symptom Severity Scale (SSS) (7), the Revised Fibromyalgia Impact Questionnaire (FIQR) (28), the 36-item Short-Form Health Survey (SF-36) (29) and the Hospital Anxiety and Depression scale (HADS) (30) were administered. These patient-reported outcome measures (PROMs) were selected to capture the subjective dimensions of functioning included in the predefined conceptual framework. Specifically, the SF-36 Physical Component was used to assess perceived physical functioning, the SF-36 Mental Component and HADS represented the emotional distress domain, whereas the WPI, SSS, and FIQ-R were used to characterize the pain impact and symptom burden domain. Together with the performance-based tests and accelerometer-derived measures, these instruments enabled a comprehensive assessment of the four predefined functioning domains based on the ICF framework.

#### 2.4.3 Performance-based tests and berg balance scale

Following questionnaires completion, participants performed four objective physical performance assessments in a standardized order:

- The HST assesses muscle power functions as described by the ICF (9, 22). Beyond self-report, the HST provides an objective measure of muscle strength that is independent of pain related reporting bias. The test was measured using a handheld calibrated dynamometer (Jamar® dynamometer, Saehan Corporation, Masan, Korea). Each participant performed the test sat on a chair, shoulder at 0° of adduction (arm against the trunk), elbow flexed to 90°, and forearm and wrist in neutral position, without support. Once positioned, participants were asked to squeeze the dynamometer and perform a maximum sustained contraction for 3 s as strong as they could. Three measurements were performed with both hands with 60-second rest periods between them to avoid fatigue (31, 32). The mean value of the three trials for each hand was noted (Kg). This test has been previously used in several studies with FM patients (12, 14, 33, 34).
- The 8FUGT is a well-established measure of overall functional mobility, encompassing changing basic body positions as described in the ICF (9, 22), as well as coordination and balance. Beyond self-reported mobility, the 8FUGT captures a composite performance outcome integrating strength, balance and coordination that patients often over or underestimate when self-reporting. It involves standing up from a chair, walking 2.44 m to and around a cone, and return to the chair in the shortest possible time without running (35). The test was performed three times, with 60-second rest periods between trials to reduce the potential impact of fatigue or pain on performance consistency. The mean time of three trials was recorded and used in the analyses (seconds). The test has demonstrated strong reliability among older adults with FM (13, 33, 35, 36).
- The 6MWT measures functional exercise capacity through cardiorespiratory fitness, corresponding to exercise tolerance functions and walking within the ICF framework (9, 22). Beyond self-reported physical functioning, the 6MWT provides an objective measure of exercise capacity that cannot be inferred from PROMs. Participants were instructed to walk safely and comfortably as quickly as possible along a 45.7 m rectangular course for six minutes. The maximum distance (in meters) walked was registered. This test has been shown good reliability in women with FM (13, 33, 37).
- The BBS is a standardized measure of functional balance that evaluates static and dynamic balance through 14 everyday functional tasks. Balance impairment is frequent in FM and usually underreported by patients; the BBS therefore provides an objective, clinically graded score not otherwise captured by the self-reported measures included in this study. Scores range from 0 to 56, with higher scores indicating better balance performance. Within the ICF framework, the BBS primarily assesses maintaining and changing basic body positions and mobility-related activities (9, 22). This test is well established in the assessment of patients with FM (24–26)

Each test was conducted following standardized protocols, with verbal encouragement provided according to predefined criteria to maintain consistency. Sufficient rest intervals were allowed between tests to prevent excessive fatigue from influencing subsequent performances.

A summary of the rationale for selecting each PBT, mapped to its corresponding ICF component, supporting evidence in FM, and expected contribution beyond PROMs, is presented in Supplementary material/Appendix 1.

#### 2.4.4 Physical activity levels and sedentary time

Participants were instructed to wear a triaxial GT3X + accelerometer (ActiGraph®, Pensacola, FL, USA) continuously for 9 consecutive days, with removal only required for water-based activities. The device was positioned at the hip and attached using an elastic belt worn beneath the clothing. Accelerometer data were sampled at 30 Hz and aggregated into 60-second epochs, in accordance with previously validated cut-point methodologies (38, 39).

Accelerometer wear time was calculated by deducting non-wear periods from the total recording time for each day. Sleep duration was derived from a sleep diary in which participants recorded their bedtime and wake-up time. Non-wear time was identified using Choi's algorithm (40), defining non-wear periods as intervals of at least 90 consecutive minutes with 0 activities counts, allowing for up to 2 min of non-zero counts (spike tolerance) provided that the preceding and subsequent 30-minute windows also registered zero counts. Default parameters implemented in ActiLife® software (Version 6.13.4) were used for this classification. To minimize potential reactivity related to participants awareness of being monitored, PA data from the first monitoring day was excluded. Additionally, data from the last day, with the return of the device, was also excluded from the analysis. Inclusion in the study required a minimum of 7 consecutive days of accelerometer recording, with at least 10 valid wear hours per day. Data download, reduction, cleaning, and analysis were conducted using the manufacturer software ActiLife® Version 6.13.4. (Actigraph).

Sedentary time was defined as the accumulated duration of wear time with activity below 200 counts per minute (39). PA intensity levels—light, moderate, vigorous, and moderate to vigorous PA (MVPA)—were derived using recommended vector magnitude cut-points (38): 200–2,689 counts/min for light PA, 2,690–6,166 counts/min for moderate, ≥ 6,167 counts/min for vigorous, and ≥ 2,690 counts/min for MVPA. These thresholds have been previously applied and validated in studies involving individuals with FM (41, 42), allowing direct comparability of PA intensity classifications with the existing FM literature. The use of vector magnitude, rather than single axis counts, additionally captures multi-planar movement patterns more sensitively, which is relevant given the altered movement and gait patterns often reported in this population All outcomes were expressed as minutes per day.

### 2.5 Conceptual framework and construct validity hypotheses

#### 2.5.1 Definition of constructs based on the ICF

The conceptual framework was developed to define the study constructs and to organize all measurement instruments into theoretically grounded domains of functioning. To further ensure conceptual consistency, all study variables (PBTs, PROMs, and accelerometer-derived measures) were mapped to categories of the ICF Comprehensive Core Set for CWP (15). Specifically, variables were linked to relevant domains within the Body Functions [e.g., exercise tolerance (b455), muscle power (b730), sensation of pain (b280)] and Activities and Participation (e.g., walking capacity (d450), changing basic body position (d410), maintaining body position (d415) and moving around (d455)) components following linking rules by Cieza et al. (22). This ICF-based mapping enabled the identification of the specific ICF categories represented by each outcome measure and provided the basis for grouping the instruments into four theoretically derived constructs: Physical functioning, activity behavior, emotional distress, and pain impact and symptom burden. These four theoretically derived constructs served as the basis for the construct validity hypotheses described in the following section.

#### 2.5.2 Construct validity hypotheses

Construct validity was evaluated using a hypothesis-driven approach based on *a priori* conceptual framework grounded in the ICF and the biopsychosocial model of FM. All study variables were theoretically mapped into four predefined constructs: (1) Physical functioning, (2) activity behavior, (3) emotional distress, (4) pain impact and symptom burden.

Physical functioning was defined as a formative construct, with indicators defining a unique part of the construct/domain instead of acting as interchangeable indicators (43). This domain was conceptualized as a composite construct integrating both objective capacity (PBTs and BBS) and perceived physical functioning (SF-36 physical component). Activity behavior comprised accelerometer-derived indicators of habitual PA and sedentary time. Pain impact and symptom burden included FIQ-R total score, WPI and SSS. Emotional distress was defined using the SF-36 mental component and HADS subscales.

Based on this framework, specific hypotheses were formulated regarding the expected relationships between domains. Convergent validity was hypothesized between conceptually related domains. Specifically, weak to moderate associations were expected between physical functioning (PBTs and perceived physical functioning) and activity behavior (r ≈ 0.25–0.40), reflecting the relationship between objective capacity and real-world performance within the same functional continuum in the ICF framework. Moderate to strong associations were expected between pain impact and symptom burden and emotional distress (r ≥ 0.50), given their shared subjective and psychosocial nature (Supplementary material/Appendix 2). Furthermore, because physical functioning represents the central construct of the proposed assessment framework, specific hypotheses were established regarding the relationships among its indicators. Moderate to strong associations (r ≥ 0.50) were expected among performance-based measures (6MWT, HST, 8FUGT, and BBS), as these instruments assess complementary dimensions of objectively measured physical capacity, including exercise tolerance, muscle strength, balance and mobility. Moderate associations (r = 0.30–0.59) were expected between objective performance measures and the SF-36 physical component, reflecting the relationship between observed functional capacity and perceived physical functioning within the broader physical functioning domain (Supplementary material/Appendix 3).

Discriminant validity was assessed by examining relationships between conceptually distinct domains. Weak or negligible correlations (r < 0.20) were expected between activity behavior and both pain impact and symptom burden and emotional distress, reflecting the relative independence of habitual activity patterns from subjective symptom appraisal. Low to moderate associations were expected between physical functioning and emotional distress (r ≈ 0.25–0.40), indicating limited overlap between objective capacity and psychological states. Importantly, although physical functioning (PBTs) and pain impact and symptom burden are conceptually distinct constructs, a moderate association (r ≈ 0.30–0.50) was hypothesized (Supplementary material/Appendix 2). This assumption is supported by previous evidence suggesting that symptoms severity and perceived disease impact can influence physical performance in FM (37, 44, 45). Therefore, this relationship was interpreted as reflecting related but non redundant constructs, rather than true convergence.

### 2.6 Statistical analysis

Descriptive statistics [mean and standard deviation (SD)] of age and anthropometric measurements were calculated for the whole sample. Categorical variables are reported as percentages.

To explore the underlying factor structure of the instrument, an exploratory factor analysis (EFA) was conducted. The suitability of the data for factor analysis was assessed using the Kaiser Meyer Olkin (KMO) measure of sampling adequacy and Bartlett's test of sphericity. Factor extraction was performed using principal axis factoring, as it does not assume multivariate normality and is appropriate for identifying latent constructs. The number of factors was determined based on eigenvalues greater than 1.0, inspection of the scree plot, and theoretical interpretability. To facilitate interpretation, an oblique rotation (promax) was applied, allowing for correlations between factors. Factor loadings ≥ 0.30 were considered meaningful. The final factor solution was selected based on a combination of statistical criteria and conceptual coherence.

Associations between PBTs, accelerometer-derived PA variables, and PROMs were examined using Spearman's correlation coefficient (rho). Correlation strength was interpreted as weak (r < 0.30), moderate (r = 0.30–0.59), or strong (r ≥ 0.60). Bootstrapped 95% confidence intervals (CI) (1,000 replications) were computed for all Spearman's rho correlation coefficients.

The statistical significance was established at the *p* ≤ 0.05 level. Statistical analysis was conducted using the Statistical Package for the Social Sciences (SPSS, version 24.0; IBM Corp., Armonk, NY, USA) and R software (version 4.4.1) as needed.

## 3 Results

### 3.1 Sample characteristics

**Figure 1:**
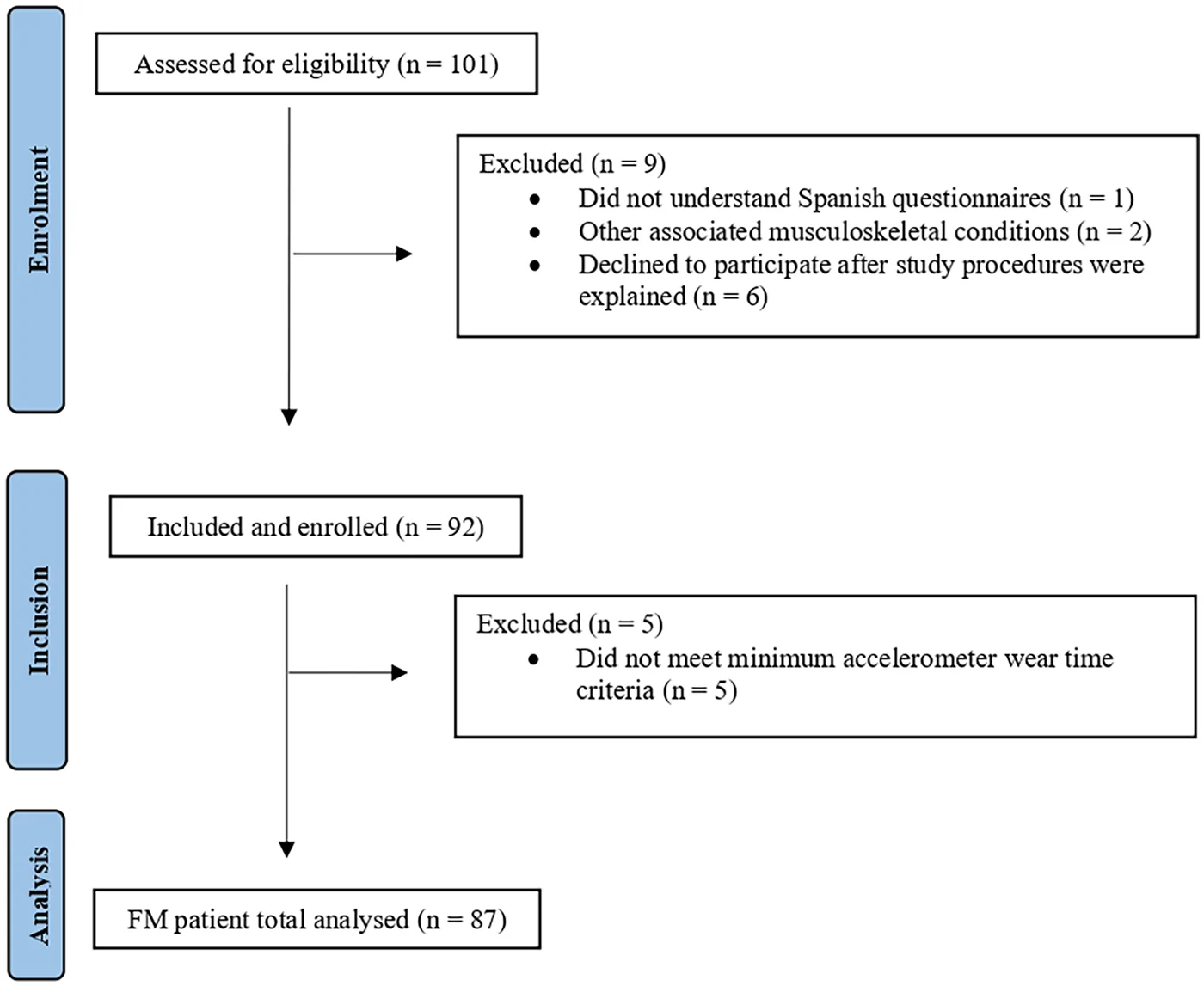
STROBE flow diagram of participant recruitment, exclusion, inclusion in the final sample.

The flow of participants through the study, from initial eligibility assessment to final analysis, is presented in Figure 1. Sociodemographic and anthropometric characteristics of the study sample are presented in Table 1. A total of 87 women with FM were included. The mean age was 52.62 years (SD = 8.11), with a mean BMI of 28.04 kg/m^2^ (SD = 5.35). Most participants were classified as overweight or obese (66.67%). The sample was predominantly of European ethnicity (97.70%). Regarding work status, the distribution was balanced across the three categories: actively working (34.48%), claiming disability (34.48%), and with recognized disability (31.04%). Most participants had completed secondary or university education (73.56%).

**Table 1:** Sociodemographic and anthropometric characteristics of the participants.

Age & Anthropometric Data	Participants (*n* = 87)
	Mean (SD)
Age (years)	52.62 (8.11)
Height (m)	1.60 (0.06)
Weight (kg)	72.05 (14.00)
BMI (kg/m^2^)	28.04 (5.35)
BMI classification (kg/m^2^)	*n* (%)
Underweight (<18.5)	2 (2.30)
Normal weight (18.5–24.9)	27 (31.03)
Overweight (≥25.0)	33 (37.93)
Obesity (≥30.0)	25 (28.74)
Ethnicity	*n* (%)
European	85 (97.70)
Latin American	1 (1.15)
Maghrebi	2 (2.30)
Work status	*n* (%)
Active	30 (34.48)
Claiming disability	30 (34.48)
Disability	27 (31.04)
Marital status	*n* (%)
Married	43 (49.43)
Not married	44 (50.57)
Educational level	*n* (%)
Basic education	15 (17.24)
Secondary studies	52 (59.77)
University	20 (22.99)

BMI, body mass index; kg, kilograms; m, meters; SD, standard deviation.

### 3.2 Performance-based tests, physical activity and patient-reported outcomes measures

Descriptive PBTs, accelerometer-derived PA variables, and PROMs are presented in Table 2. Participants walked a mean distance of 346.97 m (SD = 84.73) during the 6MWT. Mean HST was 16.16 kg (SD = 7.26) for the dominant hand and 14.83 kg (SD = 7.16) for the non dominant hand, while the mean 8FUGT completion time was 9.12 s (SD = 3.10). The mean BBS score was 51.31 (SD = 6.38).

**Table 2:** Physical performance, accelerometer metrics and PROMs characteristics of the participants.

Performance based tests	Participants (*n* = 87)
	Mean (SD)
6MWT (m)	346.97 (84.73)
Handgrip dominant (Kg)	16.16 (7.26)
Handgrip non dominant (kg)	14.83 (7.16)
8FUGT (s)	9.12 (3.10)
Berg Balance Scale (0–56)	51.31 (6.38)
Accelerometer (min/day)	Mean (SD)
Wear time	874.74 (136.23)
Sedentary time	376.88 (169.62)
Light PA	411.33 (97.26)
MVPA	37.92 (26.64)
PROMs	Mean (SD)
FIQ-R total score (0–100)	73.76 (12.91)
WPI (0–19)	14.02 (2.79)
SSS (0–12)	9.77 (1.87)
SF-36 PC (0–100)	23.02 (5.34)
SF-36 MC (0–100)	41.56 (6.69)
HADS total score (0–42)	24.31 (6.92)

6MWT, 6-minute walk test; 8FUGT, 8 foot up and go test; FIQ-R, fibromyalgia impact questionnaire revised; HADS, hospital anxiety and depression; kg, kilograms; light PA, light physical activity; m, meters; min, minutes; MVPA, moderate to vigorous physical activity; s, seconds; SD, standard deviation; SF-36 MC, 36-item short form health survey mental component; SF-36 PC, 36-item short form health survey physical component; SSS, symptom severity score; WPI, widespread pain index.

Accelerometer data showed a mean wear time of 874.74 min/day (SD = 136.23). Participants spent an average of 376.88 min/day (SD = 169.62) in sedentary behavior, 411.33 min/day (SD = 97.26) in light PA, and 37.92 min/day (SD = 26.64) in moderate to vigorous PA (MVPA).

Regarding PROMs, the mean FIQ-R total score was 73.76 (SD = 12.91), indicating a high disease impact. Mean scores were 14.02 (SD = 2.79) for the WPI, 9.77 (SD = 1.87) for the SSS, 23.02 (SD = 5.34) for the SF-36 physical component, 41.56 (SD = 6.69) for the SF-36 mental component, and 24.31 (SD = 6.92) for the HADS total score.

### 3.3 Latent structure of the multidimensional functioning framework

**Figure 2:**
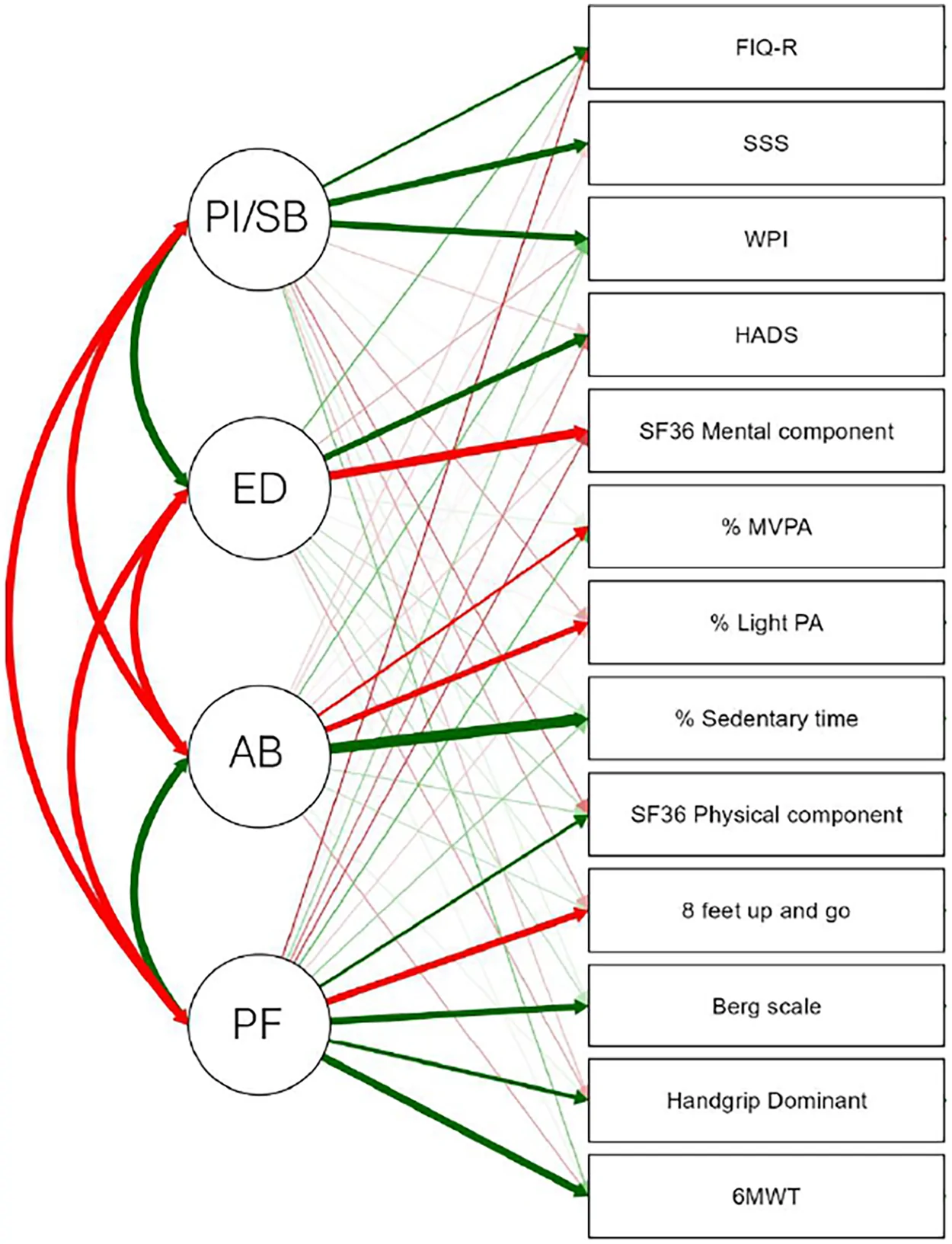
Exploratory factor analysis (EFA) structure showing the relationships between latent domains and observed variables. Green arrows indicate positive associations, whereas red arrows indicate negative associations. The intensity and thickness of the arrows reflect the strength of the factor loadings, with darker and thicker arrows representing stronger associations and lighter/thinner arrows representing weaker associations. Curved arrows between latent domains represent the correlations observed between factors. 6MWT, 6-minute walk test; AB, activity behavior; ED, emotional distress; FIQ-R, revised fibromyalgia impact questionnaire; HADS, hospital anxiety and depression scale; MVPA, moderate to vigorous physical activity; PA, physical activity; PF, physical functioning; PI/SB, pain and symptom burden; SF-36, 36-item short form health survey; SSS, symptom severity scale; WPI, widespread pain index.

To examine whether the predefined biopsychosocial domains were supported empirically, an EFA including all variables was conducted. The resulting latent structure is presented in Figure 2. Sampling adequacy was confirmed by the Kaiser Meyer Olkin measure (overall KMO = 0.629) and Bartlett's test of sphericity (*χ*^2^ (78) = 1699.93, *p* < 0.001), supporting the suitability of the data for factor analysis. The pattern matrix showed a clean simple structure, with no item exhibiting a secondary loading ≥ 0.30, so no cross-loading resolution procedure was required. The EFA identified a four-factor solution broadly consistent with the hypothesized conceptual framework, explaining 67.1% of the total variance (Table 3). The first factor represented physical functioning and accounted for 22.6% of the explained variance. This domain was characterized by strong loadings from the 6MWT (0.925), 8FUGT (−0.853), BBS (0.825), HST (0.588), and SF-36 physical component (0.486). The second factor reflected activity behavior, explaining 18.6% of the variance, with sedentary time loading positively (1.258) and light PA (−0.768) and MVPA (−0.421) loading negatively. The third factor corresponded to emotional distress, accounting for 13.3% of the variance and primarily defined by the SF-36 mental component (−1.032) and HADS (0.786). Finally, the fourth factor represented pain impact and symptom burden, explaining 12.7% of the variance, with substantial contributions from SSS (0.846), WPI (0.826), and FIQ-R (0.463).

**Table 3:** Explained variance and correlations between latent domains derived from the exploratory factor analysis.

Domains	Rotated solution			Factor correlations			
	SumSq. Loadings	Proportion var.	Cumulative	Physical functioning	Activity behavior	Emotional distress	Pain impact and symptom burden
Physical functioning	2.933	0.226	0.226	-			
Activity behavior	2.419	0.186	0.412	−0.317	-		
Emotional distress	1.728	0.133	0.545	−0.343	0.003	-	
Pain impact and symptom burden	1.645	0.127	0.671	−0.522	0.220	0.399	-

SumSq., sum of squared loadings; var., variance.

Correlations between latent domains were generally low to moderate (Table 3). The strongest association was observed between physical functioning and pain impact and symptom burden (r = −0.522), whereas activity behavior showed weak relationships with the remaining domains. emotional distress demonstrated moderate associations with both pain impact and symptom burden (r = 0.399) and physical functioning (r = −0.343), supporting the conceptual distinction but partial overlap between physical, behavioral, and psychosocial dimensions of functioning in FM.

### 3.4 ICF mapping of the study constructs

**Figure 3:**
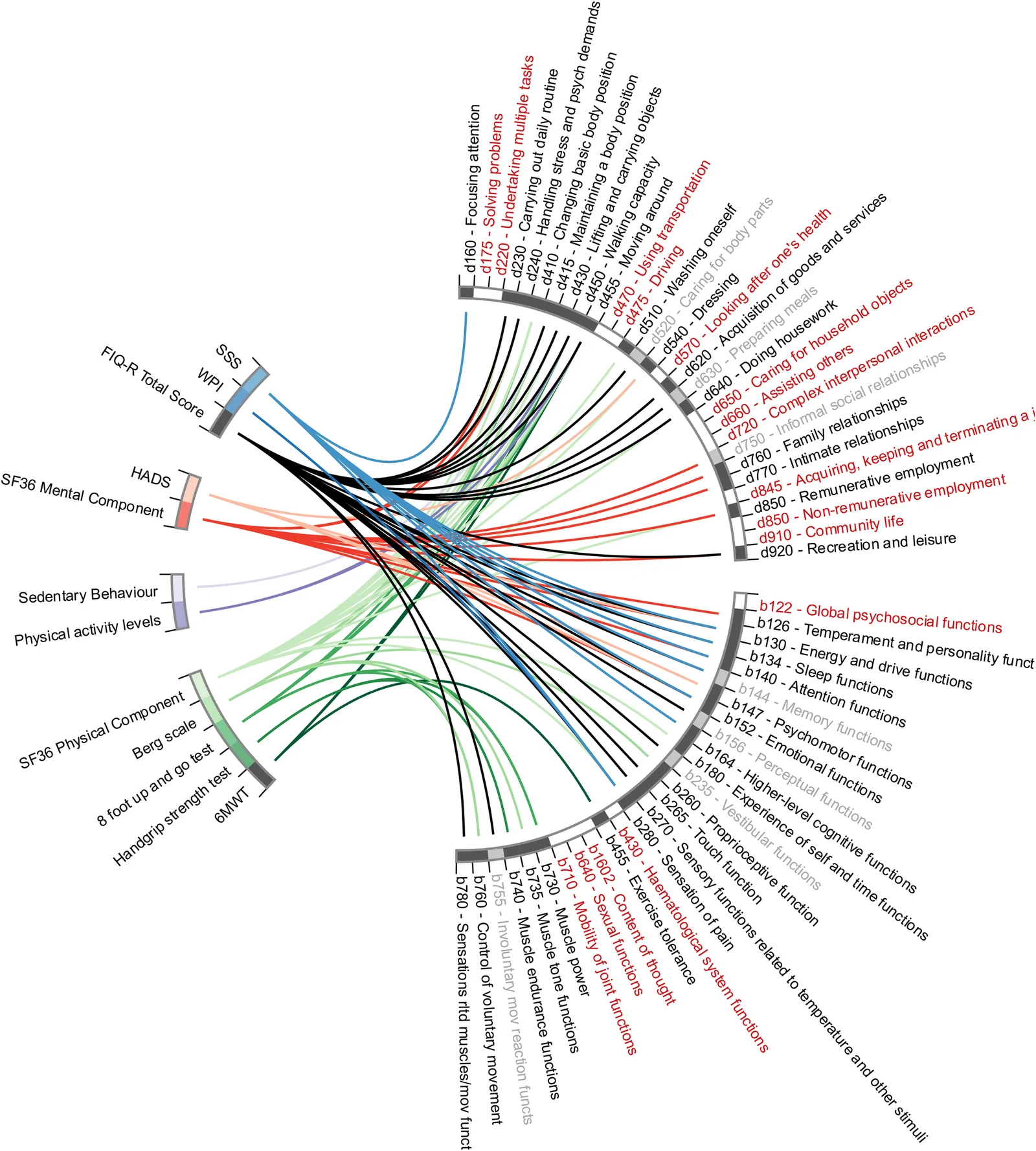
Circos plot (66) mapping the four study constructs to ICF categories. The four constructs and their respective variables are displayed on the left. Physical functioning (green), activity behavior (purple), emotional distress (red), and pain impact and symptom burden (blue). ICF categories are shown on the right, grouped into Activities and Participation (d-codes) and Body Functions (b-codes). ICF category labels are color-coded as follows: Black labels indicate categories included in the ICF Comprehensive Core Set for CWP and represented within the construct; red labels indicate Core Set for CWP categories not represented within the construct; grey labels indicate categories represented within the construct but not included in the Core Set for CWP. functs, functions; mov, movement; psych, psychological; rltd, related.

**Figure 4:**
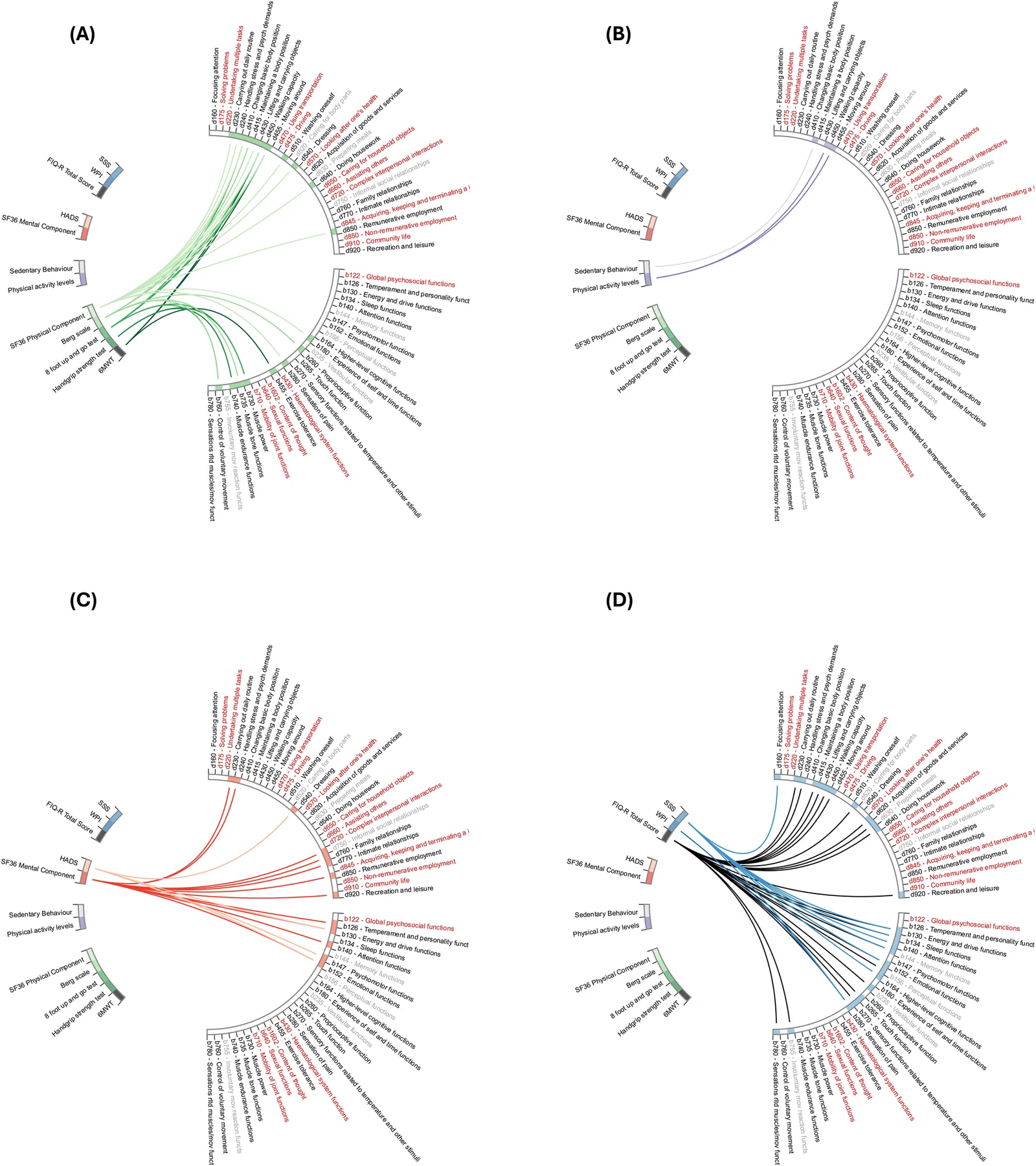
Circos plot (66) mapping the four study constructs to ICF categories. **(A)** Physical functioning, **(B)** activity behavior, **(C)** emotional distress, and **(D)** pain impact and symptom burden. Each panel illustrates the ICF categories represented by the outcome measures assigned to the corresponding construct. ICF category boxes are color-coded according to the construct to which they are linked. Categories are organized into Activities and Participation (d-codes) and Body Functions (b-codes). Black labels indicate categories included in the ICF Comprehensive Core Set for CWP and represented within the construct; red labels indicate Core Set for CWP categories not represented within the construct; grey labels indicate categories represented within the construct but not included in the Core Set for CWP. functs, functions; mov, movement; psych, psychological; rltd, related.

Circos plot (66) (Figure 3) illustrates the complete mapping between the assessment measures and the corresponding ICF categories, whereas the subsequent panels (Figure 4) display the ICF categories underpinning each construct separately, highlighting their conceptual composition.

### 3.5 Construct validity

**Figure 5:**
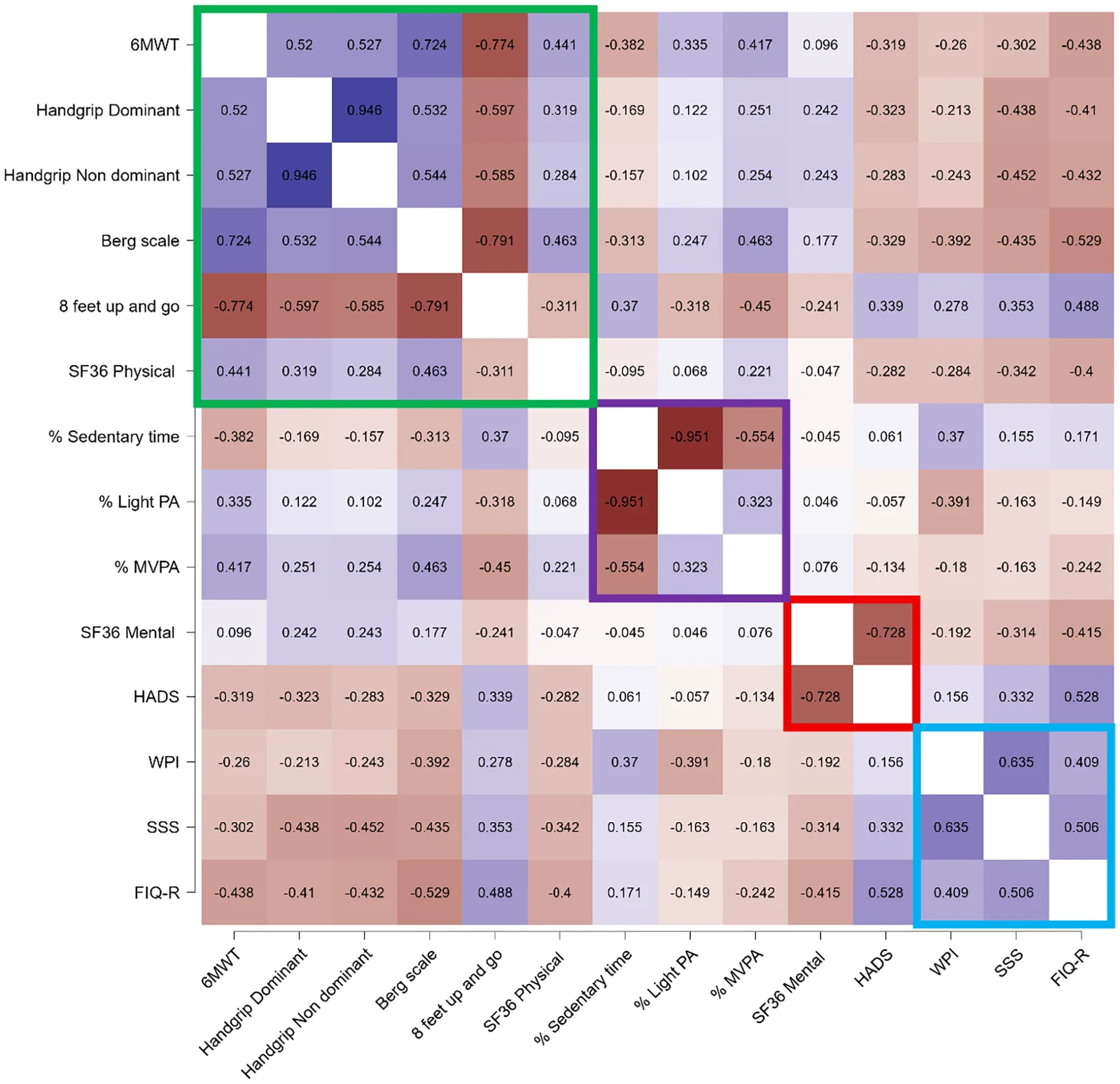
Heatmap correlation matrix across variables grouped according to the domains identified through exploratory factor analysis. Green box represents the physical functioning domain, purple box the activity behavior domain, red box the emotional distress domain, and blue box the pain impact and symptom burden domain. Cell color intensity reflects correlation strength, with darker colors indicating stronger associations. Blue tones indicate positive correlations, whereas red tones indicate negative correlations. 6MWT, 6-minute walk test; FIQ-R, revised fibromyalgia impact questionnaire; HADS, hospital anxiety and depression scale; Light PA, light physical activity; MVPA, moderate to vigorous physical activity; SF-36, 36-item short form health survey; SSS, symptom severity scale; WPI, widespread pain index.

Correlation patterns were consistent with the domains identified and supported both convergent and discriminant validity (Figure 5).

#### 3.5.1 Convergent validity

Variables within the same construct domain showed moderate to strong associations, indicating conceptual coherence between measures intended to assess related aspects of functioning and disease impact (Table 4). Within the physical functioning domain, correlations ranged from moderate to very strong. The strongest associations were observed between dominant and non dominant HST (rho (95% CI) = 0.95 (0.91; 0.97), *p* < 0.001), between BBS and the 8FUGT (rho (95% CI) = −0.79 (−0.87; −0.68), *p* < 0.001), and between the 6MWT and the 8FUGT (rho (95% CI) = −0.77 (−0.85; −0.67), *p* < 0.001). Moderate to strong correlations were also found between the 6MWT, HST, and the BBS (rho = 0.52–0.72, *p* < 0.001), and HST was also moderately and inversely correlated with 8FUGT performance (rho (95% CI) = −0.60 (−0.73; −0.42) dominant, rho (95% CI) = −0.59 (−0.72; −0.40) non dominant, both *p* < 0.001), consistent with the coordinated contribution of strength and dynamic balance to functional mobility. The SF-36 physical component showed moderate but comparatively lower associations with objective physical performance measures (rho = 0.28–0.46, *p* < 0.01), supporting its role as a complementary indicator of perceived physical functioning within the physical functioning domain.

**Table 4:** Convergent validity within each latent domain: spearman's rho correlations and 95% confidence intervals.

Pair	Spearman's rho	95% CI
Physical functioning domain		
6MWT ↔ Handgrip dominant	0.520**	(0.32; 0.68)
6MWT ↔ Handgrip non dominant	0.527**	(0.34; 0.68)
6MWT ↔ Berg Balance Scale	0.724**	(0.61; 0.81)
6MWT ↔ 8FUGT	−0.774**	(−0.85; −0.67)
6MWT ↔ SF-36 PC	0.441**	(0.23; 0.61)
Handgrip dominant ↔ Handgrip non dominant	0.946**	(0.91; 0.97)
Handgrip dominant ↔ Berg Balance Scale	0.532**	(0.35; 0.68)
Handgrip dominant ↔ 8FUGT	−0.597**	(−0.73; −0.42)
Handgrip dominant ↔ SF-36 PC	0.319*	(0.11; 0.52)
Handgrip non dominant ↔ Berg Balance Scale	0.544**	(0.37; 0.69)
Handgrip non dominant ↔ 8FUGT	−0.585**	(−0.72; −0.40)
Handgrip non dominant ↔ SF-36 PC	0.284*	(0.06; 0.49)
Berg Balance Scale ↔ 8FUGT	−0.791**	(−0.87; −0.68)
Berg Balance Scale ↔ SF-36 PC	0.463**	(0.25; 0.63)
8FUGT ↔ SF−36 PC	−0.311*	(−0.49; −0.09)
Activity behavior domain		
% Sedentary ↔ % Light PA	−0.951**	(−0.97; −0.91)
% Sedentary ↔ % MVPA	−0.554**	(−0.69; −0.37)
% Light PA ↔ % MVPA	0.323*	(0.11; 0.51)
Emotional distress domain		
SF-36 MC ↔ HADS	−0.728**	(−0.82; −0.60)
Pain impact and symptom burden domain		
WPI ↔ SSS	0.635**	(0.47; 0.75)
WPI ↔ FIQ-R	0.409**	(0.20; 0.59)
SSS ↔ FIQ-R	0.506**	(0.31; 0.67)

6MWT, 6-minute walk test; 8FUGT, 8 foot up and go test; CI, confidence intervals; FIQ-R, fibromyalgia impact questionnaire revised; HADS, hospital anxiety and depression; Light PA, light physical activity; MVPA, moderate to vigorous physical activity; rho, spearman's rank correlation coefficient; SF-36 MC, 36-item short form health survey mental component; SF-36 PC, 36-item short form health survey physical component; SSS, symptom severity score; WPI, widespread pain index.

* *p* < 0.01.

** *p* < 0.001.

Within the activity behavior domain, the strongest association was observed between % sedentary time and % light PA (rho (95% CI) = −0.95 (−0.97; −0.91), *p* < 0.001), reflecting the complementary nature of these two behaviors within the fixed daily wear time budget. % Sedentary time was also moderately and inversely correlated with % MVPA (rho (95% CI) = −0.55 (−0.69; −0.37), *p* < 0.001), whereas % light PA and % MVPA showed a comparatively weaker positive association (rho (95% CI) = 0.32 (0.11; 0.51), *p* < 0.01), consistent with these two variables capturing distinct intensity bands of movement behavior rather than a single underlying dimension. Within the emotional distress domain, a strong association was found between the SF-36 mental component and HADS (rho (95% CI) = −0.73 (−0.82; −0.60), *p* < 0.001). Within the pain impact and symptom burden domain, moderate to strong correlations were observed among WPI, SSS, and FIQ-R (rho = 0.41–0.64, *p* < 0.001). Overall, these findings support the convergent validity and internal coherence of the proposed multidimensional framework.

#### 3.5.2 Discriminant validity

Correlations between different domains were generally lower than those observed within the same domain and were mostly weak to moderate, supporting discriminant validity while preserving the expected interrelated nature of FM constructs (Figure 5). Consistent with the latent domain correlations identified in the EFA, stronger cross-domain associations were mainly observed between physical functioning and pain impact and symptom burden, as well as between emotional distress and pain impact and symptom burden, whereas activity behavior showed weaker relationships with the remaining domains. These findings support the conceptual distinction, but partial overlap, between physical, behavioral, emotional, and symptom-related dimensions of functioning in FM.

#### 3.5.3 Hypothesis confirmation rate

In accordance with COSMIN recommendations, the confirmation rate of all *a priori* formulated hypotheses was quantified (Table 5). Of the 21 predefined hypotheses—6 at the inter-domain level (Supplementary material/Appendix 2) and 15 at the item level within the physical functioning domain (Supplementary material/Appendix 3)—18 (85.7%) were fully confirmed, 2 (9.5%) were partially confirmed, and 1 (4.8%) was not confirmed. The single hypothesis not confirmed, between emotional distress and pain impact and symptom burden, was in the expected direction but below the anticipated magnitude (r = 0.399 vs. hypothesized 0.5–0.7).

**Table 5:** *A priori* hypothesis testing for construct validity: inter-domain and item-level domain relationships.

#	Pair/Domain relationship	Hypothesized r	Observed r	Direction	Outcome
Inter-domain hypotheses					
1	Activity behavior ↔ Physical functioning	≈ 0.25/0.4	−0.317	Consistent^1^	Confirmed
2	Activity behavior ↔ Pain impact and symptom burden	< 0.2	0.220	✓	Partially confirmed
3	Activity behavior ↔ Emotional distress	< 0.2	0.003	✓	Confirmed
4	Physical functioning ↔ Pain impact and symptom burden	> −0.5	−0.522	✓	Confirmed
5	Physical functioning ↔ Emotional distress	≈ −0.25/−0.4	−0.343	✓	Confirmed
6	Emotional distress ↔ Pain impact and symptom burden	≈ 0.5/0.7	0.399	✓ (below range)	Not confirmed
Item-level hypotheses within physical functioning domain					
7	Handgrip dominant ↔ Handgrip non dominant	> 0.7	0.946*	✓	Confirmed
8	Handgrip dominant ↔ 6MWT	0.5/0.7	0.520*	✓	Confirmed
9	Handgrip dominant ↔ Berg Balance Scale	0.5/0.7	0.532*	✓	Confirmed
10	Handgrip dominant ↔ SF-36 PC	0.3/0.59	0.319*	✓	Confirmed
11	Handgrip dominant ↔ 8FUGT	-0.5/−0.7	−0.597*	✓	Confirmed
12	Handgrip non dominant ↔ 6MWT	0.5/0.7	0.527*	✓	Confirmed
13	Handgrip non dominant ↔ Berg Balance Scale	0.5/0.7	0.544*	✓	Confirmed
14	Handgrip non dominant ↔ SF-36 PC	0.3/0.59	0.284*	✓ (below range)	Partially confirmed
15	Handgrip non dominant ↔ 8FUGT	−0.5/−0.7	−0.585*	✓	Confirmed
16	6MWT ↔ Berg Balance Scale	0.5/0.7	0.724*	✓	Confirmed
17	6MWT ↔ 8FUGT	−0.5/−0.7		✓	Confirmed
18	6MWT ↔ SF-36 PC	0.3/0.59	0.441*	✓	Confirmed
19	Berg Balance Scale ↔ 8FUGT	> −0.7	−0.791*	✓	Confirmed
20	Berg Balance Scale ↔ SF-36 PC	0.3/0.59	0.463*	✓	Confirmed
21	8FUGT ↔ SF-36 PC	−0.3/−0.59	−0.311*	✓	Confirmed

Of the 21 *a priori* hypotheses, 18 (85.7%) were fully confirmed, 2 (9.5%) were partially confirmed, and 1 (4.8%) was not confirmed.

6MWT, 6-minute walk test; 8FUGT, 8 foot up and go test; SF-36 PC, 36-item short form health survey physical component.

Significance levels are reported for item-level Spearman correlations only, as *p*-values are provided in standard correlation matrix output. Inter-domain (factor) correlations derive from the oblique (promax) rotation solution of the exploratory factor analysis.

1 The activity behavior factor is predominantly driven by the loading of % sedentary time; higher factor scores therefore reflect greater sedentary behavior rather than greater activity. The observed negative correlation with physical functioning is thus consistent with the hypothesized direction once this factor orientation is taken into account.

* *p* < 0.001.

## 4 Discussion

### 4.1 Construct validity of a multidimensional assessment framework

The primary objective of this study was to examine the construct validity of a predefined multidimensional assessment set of measurements, systematically including PBTs, in women with FM, following a COSMIN-guided hypothesis testing approach. Of the 21 *a priori* hypotheses formulated at the inter-domain and item level, 18 (85.7%) were fully confirmed, 2 (9.5%) were partially confirmed, and 1 (4.8%) was not confirmed, providing preliminary support for the hypothesized four-factor structure.

At the inter-domain level, 5 out of 6 hypotheses were confirmed or partially confirmed, including the expected associations linking physical functioning to both pain impact and symptom burden and emotional distress, and activity behavior to physical functioning once the sedentary time driven orientation of this factor is accounted for. The one hypothesis not confirmed, between emotional distress and pain impact and symptom burden, in the expected direction but below the anticipated magnitude (r = 0.399 vs. hypothesized 0.5–0.7), suggests that, despite their theoretical overlap, these two domains capture partially distinct aspects of the FM experience rather than a single redundant construct. This is consistent with the structural equation modeling evidence showing that psychological distress and clinical symptom severity, while related, follow distinguishable pathways in women with FM (46).

At the item level, all 15 hypotheses within the physical functioning domain were fully confirmed, supported by strong convergent relationships among physical performance measures (r = 0.28–0.95), including the expected inverse association between HST and 8FUGT performance, consistent with the coordinated contribution of muscle strength and dynamic balance to functional mobility.

Taken together, these hypothesis level findings provide support for interpreting the proposed battery as a coherent, empirically distinguishable multidimensional framework, with only the boundary between emotional distress and symptom burden identified as an area meriting further conceptual refinement in future validation work.

Previous evidence supporting the construct validity of PBTs in FM has largely focused on specific tests rather than on a comprehensive assessment framework (47). Several studies have reported correlations between the 6MWT and some PROMs such as the FIQ-R, the SF-36 physical functioning, the symptom severity or aerobic capacity, consistently showing that a better physical performance is associated with a lower disease impact and a better perceived physical function (12, 13, 37, 44). Likewise, studies evaluating HST, mobility or balance tests have demonstrated similar associations with functional status and disease severity (14, 45, 48). Although these findings are consistent with the convergent validity observed in our study, the majority of these investigations did not apply the COSMIN recommendations to evaluate construct validity, as they did not formulate *a priori* hypotheses nor distinguish between convergent and discriminant validity. Instead, they primarily reported exploratory correlations between physical performance and related clinical outcomes. Other studies have contributed to the evidence related to the known-groups validity of certain tests by demonstrating that PBTs discriminate between patients with FM and healthy controls or between different levels of disease severity, supporting their ability to reflect clinically meaningful functional differences (12, 36). However, these studies did not evaluate the broader pattern of relationships among theoretically defined constructs.

### 4.2 Exploratory factor analysis

To the best of our knowledge this is the first study showing how EFA describes the power of the present set of tests in defining symptom and disturbance clusters and their ability to capture variability. It is noteworthy that the two domains capturing the greatest amount of variability in the analyzed sample of patients with diagnosed FM were physical functioning and activity behavior. This finding strongly supports the inclusion of PBTs such as the ones proposed in the present set of measurements in patients with FM. Additionally, the second domain capturing variability was activity behavior measured by accelerometry directly supporting previous recommendations for its use in the clinical setting (11). From this, we could hypothesize that the systematic use of PBTs and actometry could be a key approach in individualizing and recording the results of the main nonpharmacological therapy in FM: exercise training (49–51). This finding is consistent with the ICF framework, in which real life performance is conceptually distinct from capacity assessed under standardized conditions. Accelerometry captures real-world activity behavior, which is influenced by contextual and behavioral factors in addition to physical functioning and symptom severity, thereby explaining its emergence as a relatively independent dimension (16).

Clinically, the four domains map onto recognizable and relevant dimensions of the FM experience: Physical functioning reflects physical capacity (e.g., assessed by standardized tests). Activity behavior reflects daily activity and participation, emotional distress reflects psychological burden, and pain impact and symptom burden mainly reflects subject perceived disability, together offering a clinically interpretable, ICF-aligned structure for individualized assessment (9).

### 4.3 ICF coverage and comprehensiveness of the proposed assessment battery

Another important finding of the present study is that the proposed PBT battery provides broad and clinically meaningful coverage of the ICF Comprehensive Core Set for CWP. The selected PBTs collectively represent several of the key Body Functions and Activities and Participation categories included in the Core Set, particularly those related to exercise tolerance (b455), muscle power (b730), changing and maintaining basic body position (d410-d415), walking capacity (d450), moving around (d455), and mobility-related activities. In contrast, emotional and symptom-related categories were mainly represented by PROMs, supporting the complementary role of objective and self-reported assessment within an ICF-based framework (15). Rather, both approaches capture different but complementary components of disability, fully aligning with the multidimensional perspective proposed by the ICF and with previous recommendations advocating multimodal assessment in chronic pain populations (48).

Importantly, no single PBT covered all relevant ICF domains. Instead, each test contributed with unique information. The 6MWT primarily captured exercise tolerance and walking capacity, the HST represented muscle power functions, whereas the 8FUGT reflected mobility, changing basic body position and dynamic balance. The BBS specifically captured balance-related components of postural control and stability, complementing the mobility information reflected by the 8FUGT. When combined, they covered a substantially wider spectrum of functioning than any individual measure alone, which strengthens the rationale for using a battery of complementary tests rather than relying on a single physical performance measure. This observation is consistent with the growing recognition that FM-related physical function is multidimensional and requires the assessment of complementary domains rather than relying on a single performance outcome (49, 50), and reinforces the evidence that reduced functional performance in FM reflects impairments across multiple physiological systems, including aerobic fitness, muscle strength and mobility, each contributing independently to disability and daily functioning (52).

### 4.4 Clinical implications for functional assessment and rehabilitation

Interestingly, despite the broad coverage achieved by the proposed battery, several categories included in the Comprehensive ICF Core Set for CWP remained unrepresented, particularly those related to domestic life, interpersonal relationships, employment, environmental factors, and several higher-level cognitive and psychosocial functions. This finding should not necessarily be interpreted as a limitation of the proposed assessment, but rather as a reflection of its intended purpose. The selected PBTs were designed to objectively quantify physical capacity, while these broader contextual domains are more appropriately evaluated using PROMs, structured interviews, or participation-based instruments. Consequently, comprehensive assessment of FM should combine objective physical performance testing with PROMs to ensure adequate coverage of the different components of functioning proposed by the ICF. This multidimensional approach is also consistent with current rehabilitation recommendations, which advocate for integrating objective physical performance with PROMs to guide individualized management of FM (49, 51).

From a clinical perspective, this combination may have important implications. PBTs provide objective, standardized and responsive measures of physical capacity that are less influenced by symptom perception or emotional state than self-reported outcomes, making them particularly valuable for monitoring functional changes over time and evaluating treatment response. This integrated assessment may also facilitate treatment planning, as different impairments identified by the PBT battery can be targeted through specific exercise modalities (e.g., aerobic, resistance or multicomponent training), which are currently recommended as first-line non-pharmacological interventions in FM. Moreover, because each PBT reflects a different component of physical functioning, clinicians may use the pattern of impairments to individualize exercise prescriptions. For example, reduced 6MWT performance may indicate impaired cardiorespiratory fitness requiring aerobic endurance training, whereas deficits in HST or functional mobility may support a greater emphasis on prescribing resistance, balance, or functional mobility exercises as part of an individualized rehabilitation program (53, 64). This individualized interpretation is consistent with current recommendations that recommend exercise as a cornerstone of FM management while emphasizing the need to tailor interventions according to patients’ functional limitations (54).

Furthermore, from a practical standpoint, the proposed battery is feasible for routine clinical use. The four PBTs can be administered in approximately 15–20 min, using low-cost, widely available equipment (a handheld dynamometer, a stopwatch, a measured walking course, and a chair/cone for the 8FUGT and BBS), requiring only brief clinician training and no specialized facilities. Patient burden is comparatively low, as tests are brief, self-paced, submaximal and interspersed with rest periods to limit fatigue. This favorable balance between comprehensiveness and feasibility supports the potential integration of the battery into routine FM assessment.

### 4.5 The added value of accelerometry in multidimensional assessment

Taken together, these findings extend beyond construct validity and have important implications for functional assessment in FM. In particular, the prominent contribution of objectively measured activity behavior to the latent structure identified in our study deserves emphasis. Accelerometer derived variables clustered into an independent construct and explained a substantial proportion of the overall variance, reinforcing that real life physical performance represents a distinct dimension of functioning that cannot be inferred solely from PBTs or PROMs (55, 56, 65).

Within this domain, the near perfect inverse correlation between % sedentary time and % light PA (rho = −0.95) was the strongest convergent association observed across all four domains, indicating that these two behaviors largely occupy opposite ends of the same daily activity continuum, whereas the comparatively weaker association with % MVPA (rho = 0.32) suggests that moderate to vigorous activity constitutes a more distinct behavioral component, less directly determined by the sedentary vs. light activity balance.

This dissociation between objectively measured activity and laboratory based physical performance is consistent with the broader distinction, embedded in the ICF framework, between capacity (what a person can do in a standardized clinical setting) and performance (what a person actually does in their everyday environment) (10, 11). PBTs capture capacity under optimal, supervised conditions, whereas accelerometry captures performance as shaped by pain related fear avoidance, activity pacing, environmental barriers, and psychosocial factors that do not operate during a brief clinical assessment (57–60). Evidence from chronic pain populations confirms that performance-based and objectively measured physical function are only moderately related and are influenced by different sets of psychosocial correlates, further supporting their conceptual non redundancy (56, 63).

From a rehabilitation monitoring perspective, this distinction is clinically meaningful: a patient may improve in PBT performance following an intervention without a corresponding increase in daily life activity, or vice versa, and relying on PBTs or PROMs alone would miss this discrepancy. Continuous or repeated accelerometry could therefore complement periodic PBT assessments by tracking whether functional gains achieved in clinic translate into sustained behavioral change at home, and by flagging early reductions in sedentary time as a proximal, sensitive marker of treatment response before slower to change PBT scores shift.

Collectively, these findings, together with the large proportion of variance explained by the activity behavior construct in our EFA, support the inclusion of accelerometry as part of routine multidimensional functional assessment to improve patient characterization, guide individualized rehabilitation planning, and monitor treatment response (54, 61, 62).

### 4.6 Limitations of the study and future research

This study has several limitations that should be acknowledged. First, the cross-sectional design does not allow causal inferences or assessment of responsiveness to change over time: therefore, the observed associations and factor structure should be interpreted as evidence of construct validity within a single assessment window rather than as proof of longitudinal stability or sensitivity to intervention. Second, the sample was composed exclusively of women with FM recruited from specialized hospital settings in Barcelona, which may restrict the generalizability of the findings to community-based patients, or individuals managed in primary care. Additionally, participants were recruited via email or telephone contact across four hospital centers, requiring interested individuals to proactively engage with the research team. This self-selection strategy may have favored patients who were more motivated or health-literate, potentially underrepresenting individuals with more severe disability or limited healthcare access. Nevertheless, the recruitment intentionally captured patients across a broad spectrum of work and disability status: actively employed (34.5%), with recognized disability (31.0%), and in the process of claiming disability (34.5%), thereby reflecting considerable heterogeneity in functional impact, chronicity, and psychosocial burden within the FM population. This diversity partially mitigates concerns about self-selection bias and supports the representativeness of the sample despite its limited size. Third this study was restricted to women with FM. The markedly lower clinical prevalence of FM in men, made it unfeasible, within the recruitment period and resources available, to obtain a male subsample of sufficient size to support statistically meaningful comparisons or inclusion in the factor-analytic model. Fourth, the factor-analytic solution supports the proposed multidimensional structure, but the boundaries between constructs were not entirely independent, which is expected in FM yet also means that the proposed domains should be interpreted as clinically related rather than strictly isolated dimensions. Fifth, comprehensive analysis focused on the coverage of the ICF core set for CWP by the available battery, but not all categories in the core set were directly represented; in particular, contextual and environmental domains, higher-level cognitive functions, and some participation-related areas were not assessed with dedicated instruments. Finally, because the study relied on a single sociocultural and healthcare context, caution is warranted when extrapolating these results to other clinical systems or populations in which work participation, disability pathways, and access to rehabilitation may differ.

Building on the limitations outlined above, several directions for future research emerge. First, longitudinal studies are needed to determine the responsiveness of the proposed battery to clinical change, establish minimal clinically important differences, and examine its predictive validity for rehabilitation outcomes. Second, extended recruitment periods and multi-region or multi-center collaboration would help determine whether the identified factor structure and construct validity generalize to male patients with FM. Third, community-based and primary care sampling strategies should be explored to improve the representativeness of future cohorts beyond specialized hospital settings, and to reduce the potential self-selection bias associated with proactive recruitment strategies.

## 5 Conclusion

The findings provide support for the proposed standardized set of measurements, including systematically PBTs, which appears to capture the complexity and substantial variability in functional impact and clinical burden among patients with FM. The inclusion of actigraphy, as recommended in previously published guidelines, offers valuable additional insights. Moreover, the identified four-domain framework supports the construct validity of integrating objective physical performance, activity behavior, and PROMs within the ICF-based biopsychosocial model of functioning. This multidimensional approach may facilitate a more comprehensive characterization of patients, improve individualized rehabilitation planning, and provide objective tools for monitoring response to exercise-based interventions, currently considered the cornerstone of non-pharmacological management in FM.

## Data Availability

The original contributions presented in the study are included in the article/Supplementary Material, further inquiries can be directed to the corresponding author.
