# Supplementary material for: The value of performance-based tests in the assessment of patients with fibromyalgia: a construct validity and comprehensiveness analysis of a standardized test battery
Source: Front Rehabil Sci. 2026 Sep 11;7:1947752. doi: 10.3389/fresc.2026.1947752 (PMC13612412; doi:10.3389/fresc.2026.1947752)

Supplementary Figure 2: Hypothesized relationships within the Physical Functioning construct

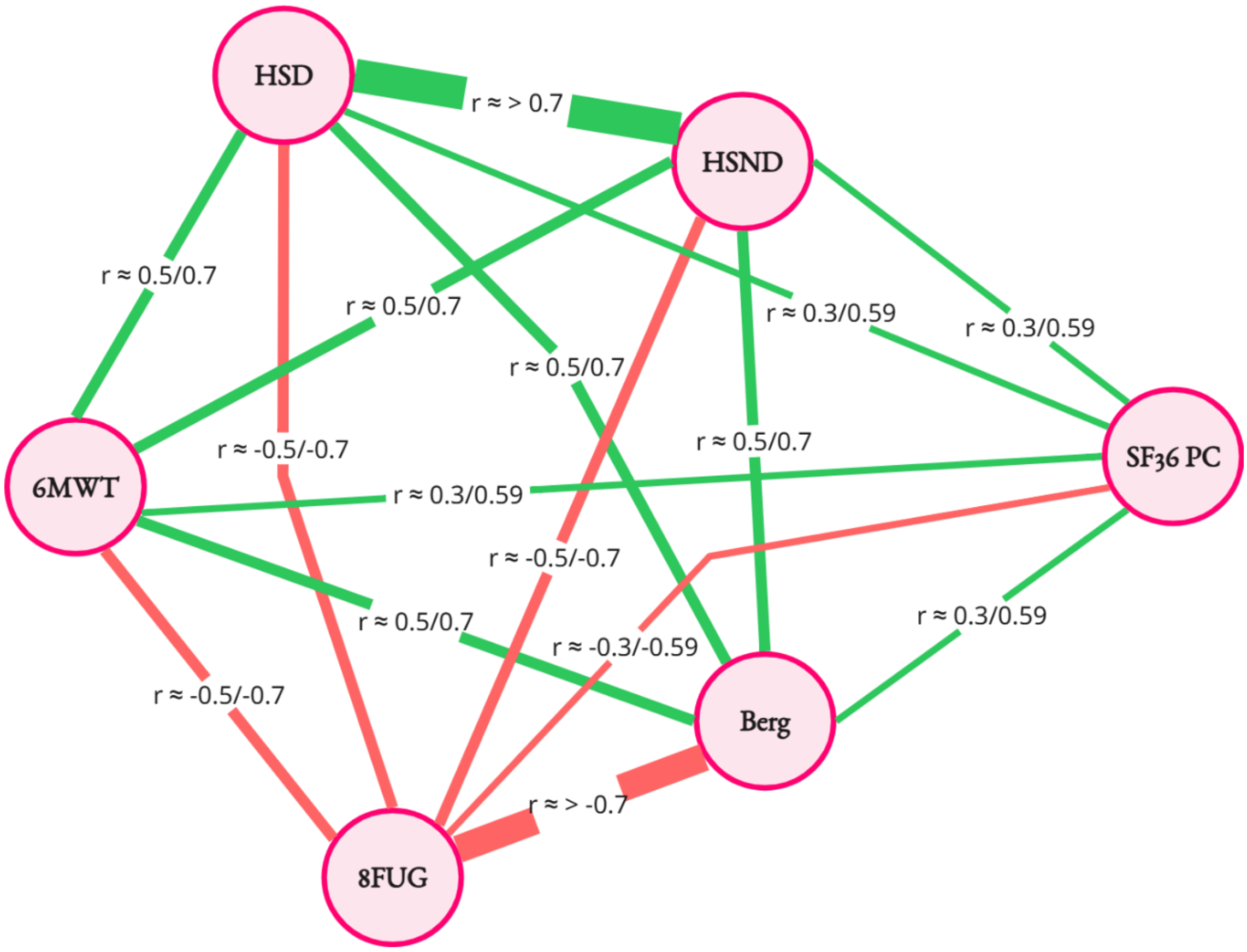

Supplement: Supplementary file 2 [file Image2.pdf]
