# Supplementary material for: The value of performance-based tests in the assessment of patients with fibromyalgia: a construct validity and comprehensiveness analysis of a standardized test battery
Source: Front Rehabil Sci. 2026 Sep 11;7:1947752. doi: 10.3389/fresc.2026.1947752 (PMC13612412; doi:10.3389/fresc.2026.1947752)
Supplement: Supplementary file 3 [file Table1.doc]

**Supplementary Table 1:** Rationale for the selection of PBTs, mapped to ICF components, supporting evidence in FM, and expected contribution beyond PROMs.

| **PBT** | **ICF component** | **Rationale for selection** | **Evidence in FM** | **Contribution beyond self-reported outcomes** |
| --- | --- | --- | --- | --- |
| 6MWT | Exercise tolerance (b455), walking capacity (d450) | Low cost, ecologically valid measure of functional exercise capacity, minimal equipment | Good reliability in women with FM (13,23,38) | Objectively quantifies cardiorespiratory/exercise capacity, not accessible via self-report |
| HST | Muscle power functions (b730) | Simple, quick, widely used strength proxy | Used in several FM studies (12,14,23,35) | Provides an objective strength measure independent of pain-related reporting bias |
| 8FUGT | Changing and maintaining basic body position (d410-d415), moving around (d455) | Integrates strength, balance and coordination in a single timed task | Strong reliability in older adults with FM (13,23,36,37) | Captures composite mobility performance often overestimated or underestimated in self-report |
| BBS | Maintaining and changing basic body position (d410-d415), moving around (d455) | Added to reinforce balance assessment, a domain not covered by the other three tests | Well established in FM assessment (26-28) | Balance impairment is frequently underreported by patients; BBS yields a graded, objective score |

Abbreviations: 6MWT, 6-Minute Walk Test; 8FUGT, 8 foot up and go test; BBS, Berg Balance Scale; FM, fibromyalgia; HST, handgrip strength test; ICF, International Classification of Functioning, Disability and Health; PBTs, performance-based tests; PROMs; patient reported outcome measures.
